# Tomato fruit as a model for tissue-specific gene silencing in crop plants

**DOI:** 10.1038/s41438-020-00363-4

**Published:** 2020-09-01

**Authors:** Ari Feder, Sarah Jensen, Anquan Wang, Lance Courtney, Lesley Middleton, Joyce Van Eck, Yongsheng Liu, James J. Giovannoni

**Affiliations:** 1 Boyce Thompson Institute for Plant Research, Cornell University, Ithaca, NY USA; 2 Section of Plant Breeding and Genetics, Cornell University, Ithaca, NY USA; 3 School of Biotechnology and Food Engineering, Hefei University of Technology, 230009 Hefei, China; 4 Section of Plant Biology, Cornell University, Ithaca, NY USA; 5 US Department of Agriculture–Agricultural Research Service, Robert W. Holley Center for Agriculture and Health, Ithaca, NY USA

**Keywords:** Plant biotechnology, Plant development

## Abstract

Use of CRISPR-Cas9 (Clustered Regularly Interspaced Short Palindromic Repeats (CRISPR)-CRISPR-associated 9)-mediated genome editing has proliferated for use in numerous plant species to modify gene function and expression, usually in the context of either transient or stably inherited genetic alternations. While extremely useful in many applications, modification of some loci yields outcomes detrimental to further experimental evaluation or viability of the target organism. Expression of Cas9 under a promoter conferring gene knockouts in a tissue-specific subset of genomes has been demonstrated in insect and animal models, and recently in *Arabidopsis*. We developed an in planta GFP (green fluorescent protein) assay system to demonstrate fruit-specific gene editing in tomato using a *phosphoenolpyruvate carboxylase 2* gene promoter. We then targeted a SET-domain containing polycomb protein, SlEZ2, previously shown to yield pleiotropic phenotypes when targeted via ^35^S-driven RNA interference and we were able to characterize fruit phenotypes absent additional developmental perturbations. Tissue-specific gene editing will have applications in assessing function of essential genes otherwise difficult to study via germline modifications and will provide routes to edited genomes in tissues that could not otherwise be recovered when their germline modification perturbs their normal development.

## Introduction

The Clustered Regularly Interspaced Short Palindromic Repeats (CRISPR)-Cas system was identified as part of the prokaryotic adaptive immune system^1^. This system has been exploited for targeted genome editing in various organisms typically via double-strand break (DSB) of targeted genomic DNA sequences via CRISPR-associated 9 (Cas9) protein directed to specific nucleotide sequences by a defined guide RNA (gRNA)^2^. High specificity and simplicity of construct development, combined with multiple adaptations of this system, has enabled an expanding range of genomic modifications in many organisms^3,4^.

In plants, heritable CRISPR-Cas9-based genomic modifications are widely deployed across numerous species^5^. These modifications can take a number of forms, including gene disruption relying upon the nonhomologous end-joining DSB repair mechanism, which is error prone leading to insertion and/or deletions (indels) mutations^5^. In addition to gene disruption, DBS can be followed by the addition of a template DNA introduced via homologous recombination (HR) to replace target sequences^6,7^. Deactivated Cas9 protein was utilized for modification of transcriptional regulation at specific loci by fusion to different effector elements^8^ as well as targeted base pair modifications using nCas9 (D10A nickase)^9^.

Spatiotemporal regulated CRISPR-Cas9 was recently reported in *Arabidopsis*^10,11^. Spatiotemporal regulation opens an additional layer for CRISPR-Cas9 application on the predicted 10% of essential genes, which are intolerant of loss or major alterations^12^. Mutations in genes involved in central processes, including primary metabolism, hormone synthesis, or signaling or epigenetic/genetic regulation, might not prove lethal, but could result in pleiotropic affects altering development or response such that assessment of gene function in a specific tissue(s) or developmental stage is not possible. An example is polycomb repressive complex 2 (PRC2), governing cell identity via histone modification-mediated gene expression changes^13,14^. Different compositions of the PRC2 complex influence multiple developmental processes throughout the plant life cycle^15^. In tomato, mutation in *SlEZ2*, a SET-domain protein of the PCR2 complex and homolog of Arabidopsis *Curly leaf* (*CLF*), exerts multiple effects on plant development. RNA interference (RNAi) *SlEZ2* plant phenotypes include dramatically reduced plant stature, abnormal flower morphology (resulting in high flower abortion rate and reduced seed number), ectopic organ development during fruit development, and reduced fruit size in addition to differences in pigmentation and cuticle properties^16,17^. Transcriptional dynamics of *SlEZ2* during fruit development suggest function in this tissue and developmental stage, but the pleiotropic effects of the mutant obstructed accurate assessment of gene function.

Given the importance of fruit biology to human food and nutritional security, we have driven CRISPR-Cas9 via a fruit-specific promoter, *PHOSPHOENOLPYRUVATE CARBOXYLASE 2* (*PPC2*), enabling generation of plants with fruit-specific gene editing inherited through the next generation. Assessment of this technology was performed through fruit-specific gene editing of a constitutively expressed *GFP* (green fluorescent protein) transgene in addition to the *SlEZ2* sequence.

## Results

**Fig. 1:**
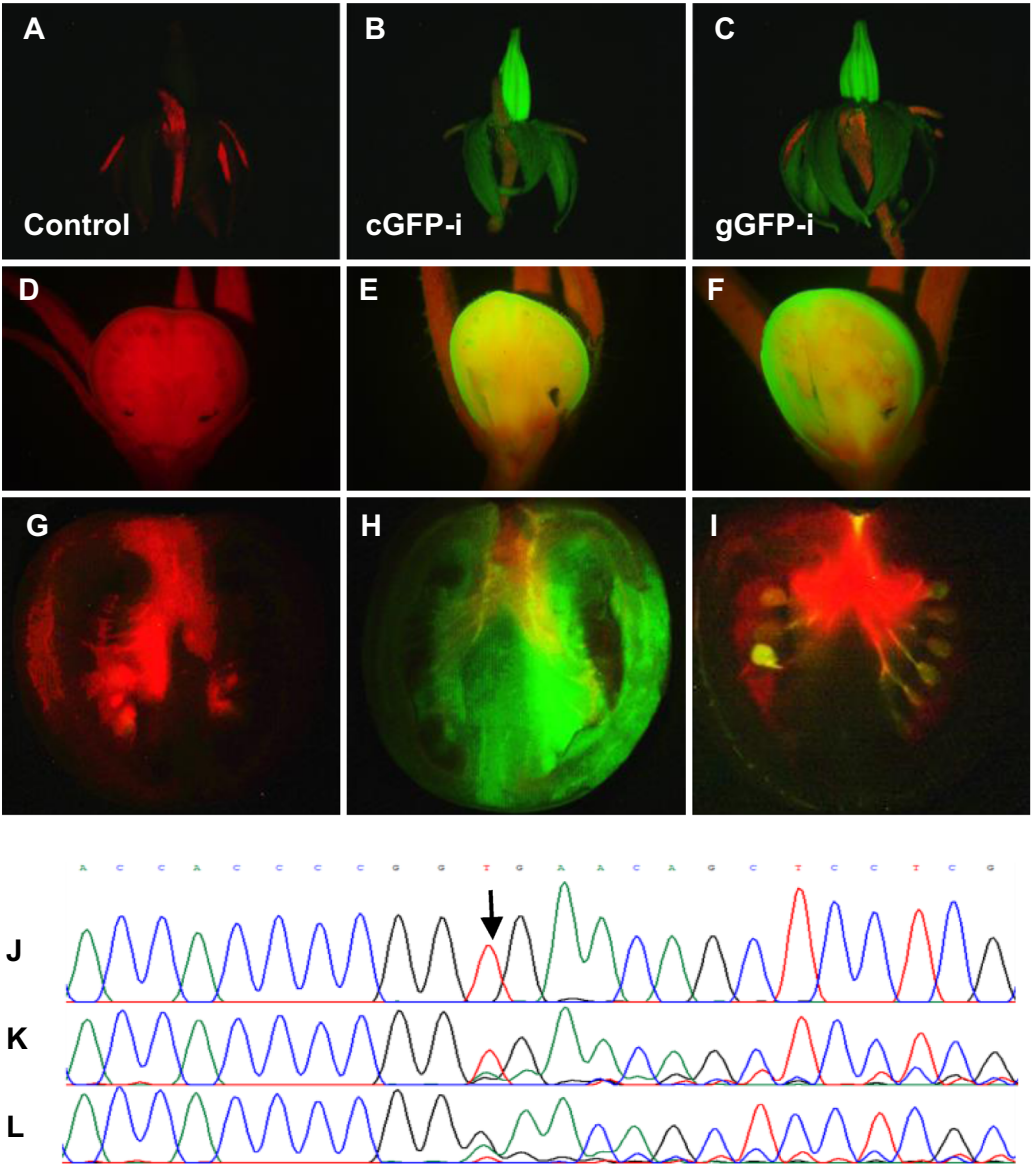
Fluorescence and sequencing of genome-integrated GFP sequence in control (untransformed) and cGFP-i or gGFP-i transgenic plants. **a**–**g** Control untransformed plants. **b**–**h** From plant transformed with a vector-expressing GFP under a constitutive promoter and Cas9 under *PPC2* promoter, without a gRNA (cGFP). **c**–**i** Plants transformed with the same construct of the GFP control, added with gRNA targeting Cas9 to GFP (gGFP). **a**–**c** Open flower, **d**–**f** fruit, 6 days after pollination, **g**–**i** fruit, breaker stage, and **j**–**l** DNA sequencing of target GFP site of **c**–**i**, respectively. Arrow indicates the Cas9 target site. Sequence shown is bases 3254–3277 of GenBank accession EF090408 of GFP.

For development of a fruit-specific gene-editing system in tomato, we wish to express Cas9 from early fruit development through ripening, while maintaining low-level expression in seeds. Toward this end, we selected the previously described *PPC2* (Solyc07g062530) gene promoter^18^. We used the p201N vector, previously shown efficient for tomato CRISPR-mediated gene silencing^19^, with two modifications: (1) the constitutive ^35^S promoter driving *Cas9* was replaced with the *PPC2* promoter and (2) *GFP* was introduced to the plasmid under the ^35^S promoter as both a marker of transgene integration and as an easily assayed reporter for testing tissue-specific editing (Fig. 1).

Initial assessment was performed by comparison of two groups of transgenic plants: (1) stably transformed with a control GFP vector (cGFP) constitutively expressing *GFP* without arming the Cas9 with a targeting gRNA, and (2) plants transformed with the same vector with the addition of a gRNA targeting Cas9 to the *GFP* sequence (gGFP vector). Three plants derived from independent transformation events of each group were analyzed. All three control cGFP-i–iii plants exhibited similar uniform GFP fluorescence throughout all plant tissues examined (Fig. 1b, e, h, Supplementary Fig. S1). The second group (gGFP-i–iii) exhibits similar fluorescence as the control cGFP lines in flowers (Fig. 1c, Supplementary Fig. S1). In contrast to the cGFP control, fruits of gGFP lines do not display GFP fluorescence during fruit development (Fig. 1i, Supplementary Fig. S1). This loss of GFP fluorescence during fruit development occurred in the context of the *GFP* gene edit at the predicted target site and was observed at early development (6 days post anthesis), where the fruit are about 3 mm in diameter (Fig. 1k, l). In contrast to elimination of GFP fluorescence in the fruit, seeds maintained fluorescence that was largely masked by the seed coat. Clear embryo and endosperm GFP fluorescence is visible in a seed bisected during slicing of the fruit (Fig. 1i). This observation was confirmed both by relative low expression of *PPC2* in seeds (http://tea.solgenomics.net/) and by further analysis of gGFP T1 segregants, where 81 and 78 T1 seedlings derived from gGFP-i and gGFP-ii plants, respectively, were analyzed (Supplementary Fig. S2). GFP fluorescence was detected in 48/81 and 66/78 from gGFP-i-T1 and gGFP-ii-T1 seedlings, respectively (Supplementary Fig. S2A), indicating that the majority of T1 seedlings inherit functional (unedited) GFP sequence. Sanger sequencing of *GFP* in 13 seedlings from gGFP-i-T1 and gGFP-ii-T1 exhibiting GFP fluorescence (Supplementary Fig. S2B, C) showed 15.4% gGFP-i and 7.7% gGFP-ii plants to be heterozygous for edited (nonfunctional) and non-edited (functional) GFP sequence, confirming that most T1 seedlings harbored unedited target *GFP* sequence. Of the 33/81 gGFP-i, and 12/78 gGFP-ii nonfluorescent T1 seedlings, 17 and 4, respectively, were PCR positive for Cas9 consistent with the presence of edited (nonfunctional) *GFP* alleles. *GFP* editing was specifically confirmed by sequencing of 11 nonfluorescent T1 seedling genomes and all had *GFP* sequence edits (Supplementary Fig. S2D). In summary, the majority of T1 plant seedling genomes inherited intact *GFP* alleles due to the low expression of PPC2-driven Cas9 expression in seed and seedlings consistent with the predominant fruit genome *GFP* disruption resulting from primary PPC2 activity in this plant organ. In order to determine consistency of fruit-specific gene silencing across generations, a T1 seedling (gGFP-i-T1–11) exhibiting GFP fluorescence (i.e., non-edited in non-fruit tissues) was grown and examined. First, the plant was confirmed to harbor non-edited *GFP* target sequences in non-fruit tissues by genomic DNA sequencing (Supplementary Fig. S2B). GFP fluorescence was then analyzed in breaker stage fruit showing that gGFP-i-T1–11 lacked fluorescence similar to the T0 plants (Fig. 1, Supplementary Fig. S3), confirming that *GFP* had been edited. Fruit from cGFP-i-T1, a plant where editing had not occurred, is shown as a positive fluorescence control.

Targeting the *GFP* transgene showed efficient gene editing in fruits via *PPC2* driven CAS9 (Fig. 1) and confirmed use of this promoter as a potential tool for studying fruit function of genes essential to additional aspects of plant development and that might yield pleiotropic phenotypes hindering functional analysis if the genome is edited in all tissues. *SlEZ2* was selected for fruit-specific gene editing due to its transcriptional dynamics during fruit development, expression in seed, and the broad range of phenotypic effects observed in *SlEZ2* RNAi making assessment of the role of SlEZ2 in fruit and seed development challenging^16^. Plants were transformed with the cGFP plasmid, including two gRNAs targeting Cas9 to *SlEZ2*. The resulting DNA construct was named gSlEZ2. Three transformed plants, gSlEZ2-i–iii, exhibiting uniform *GFP* expression and originating from independent transformation events where selected. No vegetative, floral morphology or fruit set phenotypic differences were observed when comparing the gSlEZ2 plants to cGFP controls. DNA sequencing of the two Cas9 target sites of *SlEZ2* in leaves and breaker stage fruits of the gSlEZ2 lines revealed no sequence alternation in leaves of the T0 transgenic plants at the *SlEZ2* target site, while fruit *SlEZ2* sequences were altered (Supplementary Fig. S4).

**Fig. 2:**
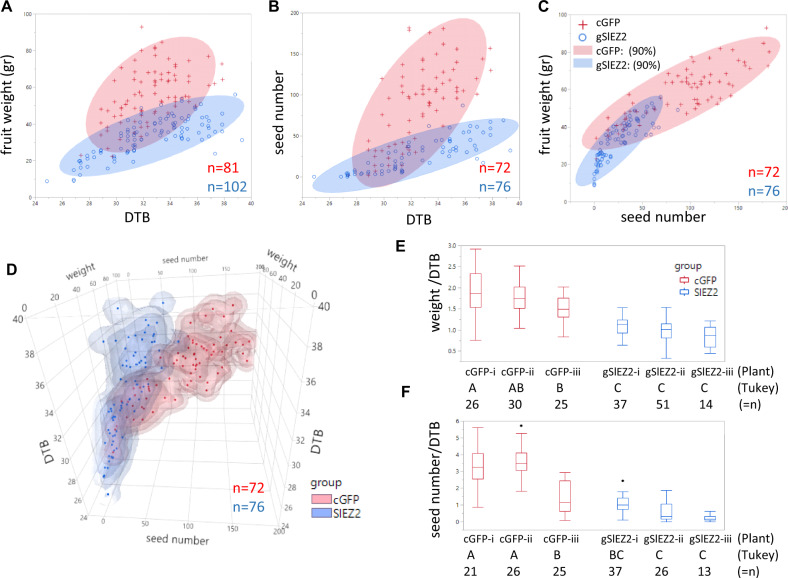
Fruit measurements of SIEZ2 and control cGFP plants. **a**–**c** Bivariate normal density ellipse plot of two groups, each consists of three stably transformed plants, of the control cGFP plants (red), and SIEZ2 plants (blue), showing the relations of fuit weight/DTB (**a**), seed number/DTB **b**, and fruit weight/seed number (**c**). **d** Three-dimensional graph of fuit showing the ratio of fruit weight to DTB (**e**) and seed number to DTB (**f**) of the individual plants within each group. Below each plot unmatching letters represent significant difference between the plants using Tukey’s HSD test (*α* 0.01). *n* represents the number of fruits tested.

Unlike previously reported *SlEZ2* RNAi fruit^16^, fruit from lines carrying the gSlEZ2 vector and with gene edited fruit exhibited normal fruit set and development. Given gene expression data indicating a role in early fruit development and/or the ripening transition^16^ (Supplementary Fig. S5), phenotyping focused on three fruit ripening and seed parameters: days from anthesis to breaker (DTB), final fruit size, and seed number. No significant change in seed weight was observed in response to loss of fruit *SlEZ2* function (Supplementary Table S2). However, fruit size and seed number varied significantly between the cGFP and gSlEZ2 plants with both substantially reduced in gSlEZ2 lines, while no significant difference was observed in DTB between these two groups (Fig. 2a–c, Supplementary Fig. S6). In addition, these results suggest that ripening time is positively associated with seed number. The three-dimensional plot in Fig. 2d depicts overall alternation in fruit development dynamics in gSlEZ2 plants as represented by seed size, fruit weight, and DTB. Demonstrating alternation in seed number correlated positively with fruit weight and DTB compared in cGFP fruit, in addition to a role of SlEZ2 in this dynamics. Checking individual plant variance of the ratio of fruit weight/DTB, and seed number/DTB showed all SlEZ2 edited lines exhibited statistically significant reduction in these ratios, except for seed number/DTB ratio of cGFP-iii compared to SlEZ2-i (Fig. 2e, f). While gSlEZ2 fruit ripens in a similar time course as cGFP, they reach this developmental stage with lower fruit mass and seed number (Fig. 2, Supplementary Fig. S6).

## Discussion

We have developed and demonstrate fruit-specific CRISPR-Cas9-based gene editing for the study of genetic contributors to fleshy fruit development. Assessment of this system was performed in tomato both with an easily monitored *GFP* transgene and the endogenous *SlEZ2* gene. CRISPR-Cas9-mediated disruption of *GFP* driven by the *PPC2* promoter showed fruit specificity in targeted disruption (Fig. 1), while enabling most transgenic T1 segregants to inherit active *GFP* sequences (Supplementary Fig. S2). PPC2-driven fruit gene editing can be efficiently performed and coupled to development of transgene seeds, which after seedling sequencing can be determined either to harbor non-edited (functional) target gene for maintenance of fruit specificity in successive generations, in addition to edited (nonfunctional alleles) for analysis of germline mutations. In addition, targeting tomato *SlEZ2*, a gene whose RNAi repression resulted in pleiotropic phenotypes^16^, enabled analysis of gSlEZ2 fruit-specific phenotypes absent of any influence of gene loss in other tissues (Fig. 2, Supplementary Figs. S4 and S6). gSlEZ2 fruit analysis revealed reductions in mass and seed number, consistent with observations from SlEZ2 RNAi^16^. In addition to fruit mass and seed number changes, *SlEZ2* RNAi fruit exhibited altered fruit shape, reduced lycopene accumulation, modified cuticle composition, and ectopic organ development^16^. It is noteworthy that no differences were observed in our fruit-specific *SlEZ2* edited lines as compared to cGFP controls with regard to fruit shape, color, texture, or ectopic organ development. We interpret these differences between fruit-specific editing and whole plant RNAi repression to mean that these traits may be regulated by *SlEZ2* in a developmental stage or tissue type prior to *PPC2* promoter expression.

While the majority of gGFP seeds inherited unedited target sequences, the *PPC2* promoter showed leakiness in the seed as revealed by occurrence of T1 gGFP seedlings harboring edited (nonfunctional) *GFP* alleles (Supplementary Fig. S2). Reduced seed number was also observed in gSlEZ2 fruit, and in the SlEZ2 RNAi antisense^16^. The basis of this phenomenon was more difficult to determine in the RNAi lines due to abnormal flower morphology and resulting decreased self-pollination efficiency. Using the fruit-specific *PPC2* promoter suggests that reduction in seed number is due to germline editing in gSlEZ2. This is further supported by the observation of parathenocarpic fruit development associated with downregulation of another PRC2 complex member, *SlFIE*^20^. As we have shown here, the decrease in seed number correlates with the decrease in fruit weight observed in gSlEZ2 fruit (Fig. 2), suggesting a linkage between these traits as shown here in our non-edited controls and has been reported previously^21,22^.

In control cGFP fruit, both seed number and fruit weight positively correlated with DTB (Fig. 2). The positive relationship between these three factors indicates that seed number regulates fruit size, which in turn is known to affect ripening time course^21,22^. Although seeds are clearly not the only factors regulating fruit size^23^, the relation between seeds to fruit size is well established^24^. In general, fleshy fruit development is accompanied by a continuant cross-talk between the developing seed with its surrounding fruit tissue. In tomato, this cross-talk involves the rate of fruit cell division, mediated by hormonal signaling from the developing embryo^25^, affecting final fruit size. Several genes have been shown to affect both seeds and fruit size^26^. Although clearly chromatin remodeling underlines cross-talk between seed and fruit development^27^, to date no remodeling mechanism has been implicated. Involvement of SlEZ2 in seed development alone does not explain changes in fruit ripening dynamics (Fig. 2), as gSlEZ2 exhibit relative delay in ripening when compared to cGFP fruit with similar seed and weight, suggesting SlEZ2 involvement in the regulation of seed-dependent fruit ripening dynamics (Fig. 2). In addition to the previously described model of SlEZ2 participation at early fruit developmental stages^17^, our results indicate that this gene influences later fruit development as well. Consistent with this interpretation is the gene’s expression decline in multiple fruit tissues ripening stages (Supplementary Fig. S5).

In summary, we show fruit-specific gene silencing of a readily monitored GFP transgene and the endogenous *SlEZ2* gene in tomato. T1 analysis revealed that most seedling genomes inherit non-edited *GFP*, indicating that the fruit specificity of the system can be maintained in T1 plants due to low activity of the *PPC2* promoter in seeds. Comparison of *SlEZ2* silencing to previous whole plant RNAi-mediated silencing both clarified the interpretation of prior results and suggests an additional role in fruit maturation. The fruit genome editing platform presented here should prove an effective tool facilitating the bypass of developmental pleiotropic effects of genes involved in fruit biology.

## Materials and methods

### Vector construction

p201N-Cas9 plasmid^28^ was used as a backbone, added with CaMV35S-sGFP(S65T)-Nos amplified from pJL33 vector^29^ using GFP-F/R primers, which was entered to p201N-Cas9 at *Pac*I-digested site, using In-fusion kit (Clontech), resulting in p201N-Cas9-GFP plasmid. Replacing the ^35^S promoter of Cas9 with the fruit-specific PPC2, in order to obtain the cGFP vector, p201N-Cas9-GFP was digested with *Swa*I and *Blp*I restriction enzymes (linearizing vector, but also cutting out a fragment from Cas9 coding region), and three fragment assembly was performed using NEBuilder HiFi DNA Assembly Master Mix (New England Biolabs) of the following fragments: linearized plasmid, Cas9 cut fragment (amplified with CasFrag-F/R primers), and PPC promoter (amplified with PPC-F/R primers). During this process the *Swa*I–*Spe*I vector linearizing sites were replaced with *Mfe*I–*Avr*II, and in addition, an *Stu*I restriction site was added at the end of the PPC2 promoter, enabling a future convenient promoter replacement if desired. Final cGFP sequence is indicated in Supplementary File S1. For assembling the SlEZ2 vector, first each gRNA was assembled separately with its own promoter and scaffold: SlEZg1 oligo, containing the first gRNA, was assembled with MtU6 promoter (PCR amplified from PCU gRNA vector^28^ using *Mfe*I_MtU6-F/MtU6-R primers) and scaffold (PCR amplified from PCU gRNA vector^28^ using Scaffold-F/UNS1_Scaffold-R primers) using NEBbuilder, followed by PCR amplification using *Mfe*I_MtU6-F/UNS1_Scaffold-R primers. In a similar manner, SlEZg2, containing the second gRNA, was assembled with MtU6 promoter (UNS1_MtU6-F/MtU6-R primers) and scaffold (Scaffold-F/*Avr*II_Scaffold-R primers) and PCR amplified with UNS1_MtU6-F/*Avr*II_Scaffold-R primers. Final vector was constructed through assembly of these two gRNA containing PCR products with the *Mfe*I/*Avr*II-digested cGFP, using NEBbuilder (New England Biolabs). Construction of the gGFP vector was performed in a similar manner, replacing the gRNA containing oligos to GFPg1/2. Final assembly of the three fragments followed incorporation of only a single gRNA (gGFP2). gRNAs were designed using The CCTop—CRISPR/Cas9 target online predictor, using target motif of GN19(NGG). Target gene sequencing was performed with GFPseqF/R, SlEZseq-F1/R1, and SlEZseq-F2/R2. Oligo sequences indicated in Supplementary Table S1.

### Plant transformation, growth, and measurements

Generation of transgenic plants was performed according to ref. ^30^, in the background of cv. Ailsa Craig. Transgenic plants were selected by uniform GFP fluorescence. Plants were moved from culture to acclimation in a growth chamber (16 h light, 24/20 °C day/night). After acclimation, plants were moved to the Guterman Bioclimatic Laboratory, Cornell University. Day temp. 21–24 °C, night 16–21 °C, and 14 h illumination. Fruits were tagged at 1 cm during July–August 2017. Fluorescence was analyzed using Olympus SZX-12 Stereo Microscope, Filter Cube LP Green: Ex. 470/40, Em. LP 500. Pictures were taken using ProgRes C14 camera (Jenoptic).

## Supplementary information

Supplemental Figure 1

Supplemental Figure 2

Supplemental Figure 3

Supplemental Figure 4

Supplemental Figure 5

Supplemental Figure 6

Supplemental File 1

Supplemental Tables S1-S2

## References

[1] Barrangou R (2007). CRISPR provides acquired resistance against viruses in prokaryotes. Science.

[2] Jinek M (2012). A programmable dual-RNA-guided DNA endonuclease in adaptive bacterial immunity. Science.

[3] Adli M (2018). The CRISPR tool kit for genome editing and beyond. Nat. Commun..

[4] Barrangou R, Doudna JA (2016). Applications of CRISPR technologies in research and beyond. Nat. Biotechnol..

[5] Demirci Y, Zhang B, Unver T (2018). CRISPR/Cas9: an RNA-guided highly precise synthetic tool for plant genome editing. J. Cell Physiol..

[6] Dahan-Meir T (2018). Efficient in planta gene targeting in tomato using geminiviral replicons and the CRISPR/Cas9 system. Plant J..

[7] Li J (2016). Gene replacements and insertions in rice by intron targeting using CRISPR–Cas9. Nat. Plants.

[8] Piatek A (2015). RNA-guided transcriptional regulation in planta via synthetic dCas9-based transcription factors. Plant Biotechnol. J..

[9] Kang B-C (2018). Precision genome engineering through adenine base editing in plants. Nat. Plants.

[10] Decaestecker, W. et al. CRISPR-TSKO: a technique for efficient mutagenesis in specific cell types, tissues, or organs in arabidopsis. *Plant Cell*10.1105/tpc.19.00454 (2019). 10.1105/tpc.19.00454 PMC6925012 31562216

[11] Liang Y (2019). A screening method to identify efficient sgRNAs in *Arabidopsis*, used in conjunction with cell-specific lignin reduction. Biotechnol. Biofuels.

[12] Lloyd JP, Seddon AE, Moghe GD, Simenc MC, Shiu SH (2015). Characteristics of plant essential genes allow for within- and between-species prediction of lethal mutant phenotypes. Plant Cell.

[13] Kohler C, Hennig L (2010). Regulation of cell identity by plant Polycomb and trithorax group proteins. Curr. Opin. Genet Dev..

[14] Pu L, Sung ZR (2015). PcG and trxG in plants—friends or foes. Trends Genet..

[15] Xiao J, Wagner D (2015). Polycomb repression in the regulation of growth and development in *Arabidopsi*s. Curr. Opin. Plant Biol..

[16] Boureau L (2016). A CURLY LEAF homologue controls both vegetative and reproductive development of tomato plants. Plant Mol. Biol..

[17] Bucher, E., Kong, J., Teyssier, E. & Gallusci, P. in *Advances in Botanical Research*, Vol. 88 (eds Mirouze, M., Bucher, E. & Gallusci, P.) 327–360 (Academic Press, 2018).

[18] Fernandez AI (2009). Flexible tools for gene expression and silencing in tomato. Plant Physiol..

[19] Jacobs TB, Zhang N, Patel D, Martin GB (2017). Generation of a collection of mutant tomato lines using pooled CRISPR libraries. Plant Physiol..

[20] Liu DD, Dong QL, Fang MJ, Chen KQ, Hao YJ (2012). Ectopic expression of an apple apomixis-related gene MhFIE induces co-suppression and results in abnormal vegetative and reproductive development in tomato. J. Plant Physiol..

[21] Chakrabarti M (2013). A cytochrome P450 regulates a domestication trait in cultivated tomato. Proc. Natl Acad. Sci. USA.

[22] Gouthu S, Deluc LG (2015). Timing of ripening initiation in grape berries and its relationship to seed content and pericarp auxin levels. BMC Plant Biol..

[23] Tanksley SD (2004). The genetic, developmental, and molecular bases of fruit size and shape variation in tomato. Plant Cell.

[24] Nitsch JP (1953). The physiology of fruit growth. Annu. Rev. Plant Physiol..

[25] Gillaspy G, Ben-David H, Gruissem W (1993). Fruits: a developmental perspective. Plant Cell.

[26] Azzi L (2015). Fruit growth-related genes in tomato. J. Exp. Bot..

[27] Zhong S (2013). Single-base resolution methylomes of tomato fruit development reveal epigenome modifications associated with ripening. Nat. Biotechnol..

[28] Jacobs TB, LaFayette PR, Schmitz RJ, Parrott WA (2015). Targeted genome modifications in soybean with CRISPR/Cas9. BMC Biotechnol..

[29] Floss DS, Levy JG, Levesque-Tremblay V, Pumplin N, Harrison MJ (2013). DELLA proteins regulate arbuscule formation in arbuscular mycorrhizal symbiosis. Proc. Natl Acad. Sci. USA.

[30] Van Eck, J., Keen, P. & Tjahjadi, M. in *Transgenic Plants: Methods and Protocols* (eds. Kumar, V., Barone, P. & Smith, M.) 225–234 (Springer, New York, 2019).

