# Supplementary figures and images for: Tomato fruit as a model for tissue-specific gene silencing in crop plants
Source: Hortic Res. 2020 Sep 1;7:142. doi: 10.1038/s41438-020-00363-4 (PMC7459100; doi:10.1038/s41438-020-00363-4)

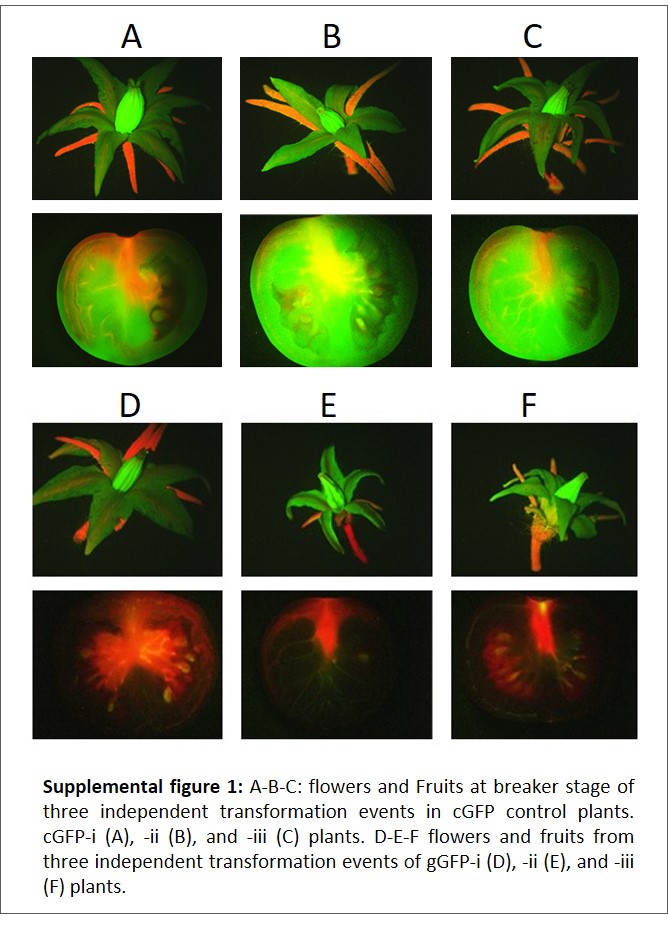

Supplement: Supplementary file 1 — Supplemental Figure 1 [file 41438_2020_363_MOESM1_ESM.jpg]

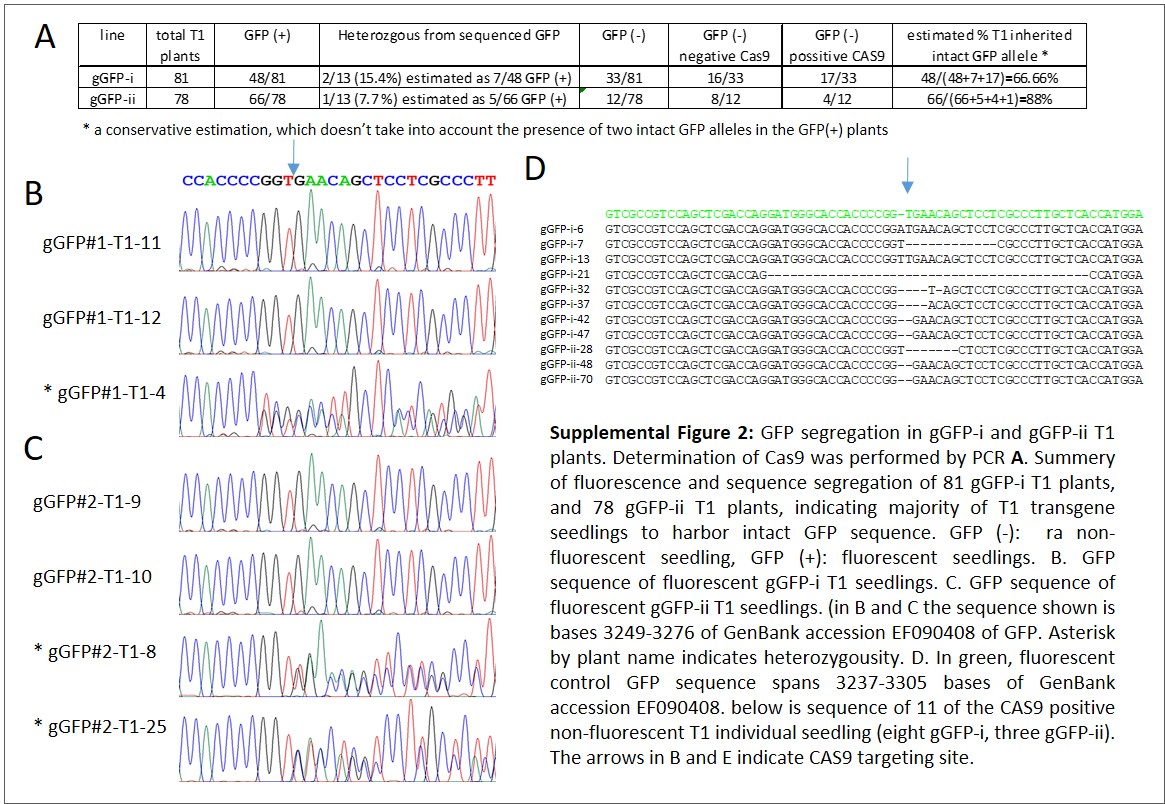

Supplement: Supplementary file 2 — Supplemental Figure 2 [file 41438_2020_363_MOESM2_ESM.jpg]

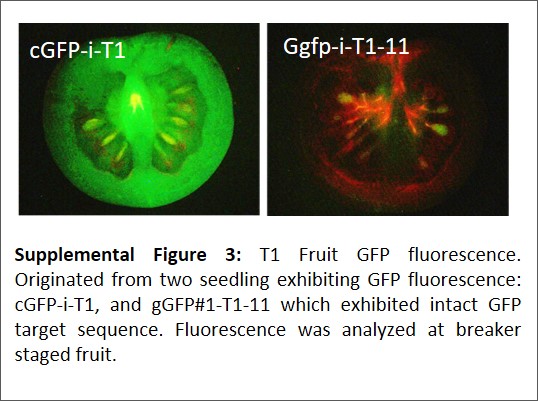

Supplement: Supplementary file 3 — Supplemental Figure 3 [file 41438_2020_363_MOESM3_ESM.jpg]

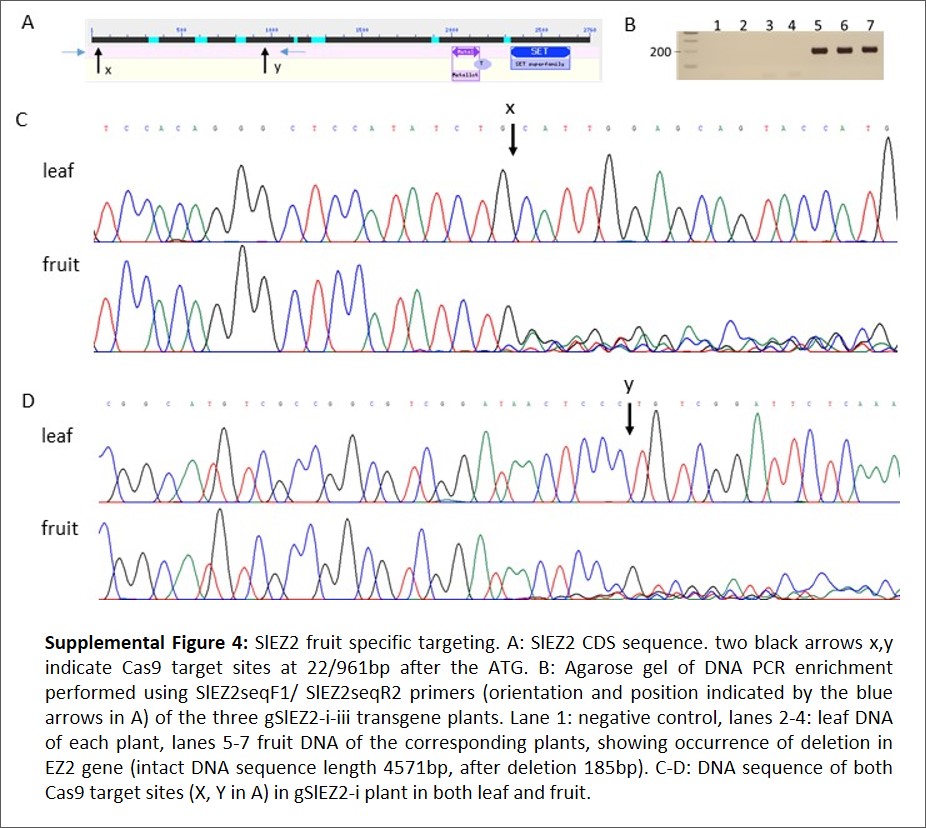

Supplement: Supplementary file 4 — Supplemental Figure 4 [file 41438_2020_363_MOESM4_ESM.jpg]

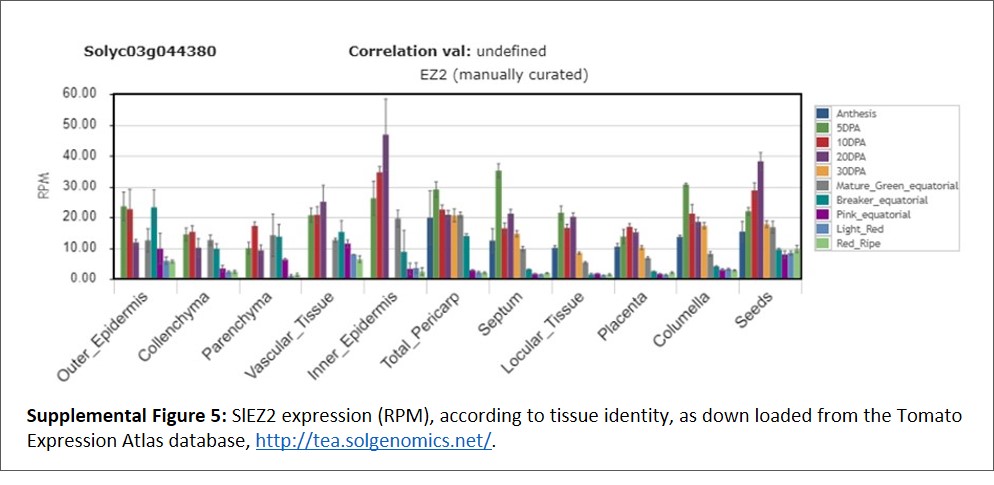

Supplement: Supplementary file 5 — Supplemental Figure 5 [file 41438_2020_363_MOESM5_ESM.jpg]

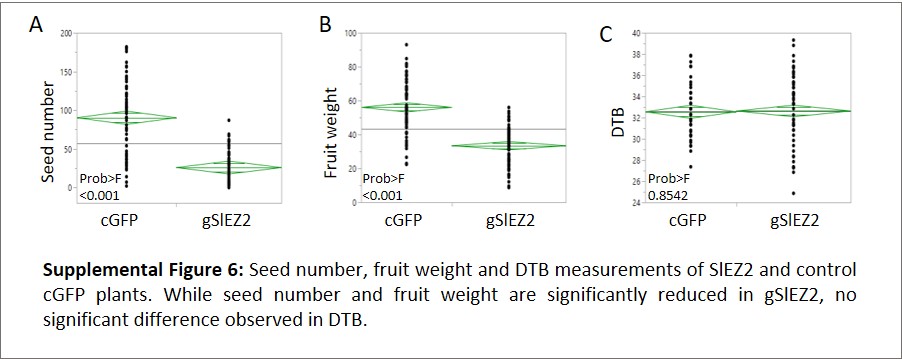

Supplement: Supplementary file 6 — Supplemental Figure 6 [file 41438_2020_363_MOESM6_ESM.jpg]
