# Supplementary material for: Tomato fruit as a model for tissue-specific gene silencing in crop plants
Source: Hortic Res. 2020 Sep 1;7:142. doi: 10.1038/s41438-020-00363-4 (PMC7459100; doi:10.1038/s41438-020-00363-4)
Supplement: Supplementary file 8 — Supplemental Tables S1-S2 [file 41438_2020_363_MOESM8_ESM.pdf]

Supplemental table 1: Oligo sequences

name	sequence
GFP-F	GGTAGGCGCGGCTTAATTAAGAATTCTCATGTTTGACAGCTTATC
GFP-R	GTACGGCGCGGCTTAATTAATGAAGGCGGGAAACGAC
PPC-F	TAATCTCTTCGATGAAATTTCAATTGCGATAACTATGTGCTTTGGATCGATCTGCCCTAGGATACATTCTACTTTGAAGTTGTTTAATG
PPC-R	TTGTCCATGCTAGCAGGCCTAACCCCCTTTTTACTCAAACTA
CasFrag-F	AGGCCTGCTAGCATGGACAAGAAGTACTCC
CasFrag-R	ATCATAGCGCTTGATCATACTAGCGCTCAGC
MfeI-MtU6-F	GATATTAATCTCTTCGATGAAATTTCATGCCTATCTTATATGATCAATGAGG
MtU6-R	AAGCCTACTGGTTCGCTTGAAG
scaffold-F	GTTTTAGAGCTAGAAATAGCAAGTT
AvrII-Scaffold-R	CATTAAACAACTTCAAAGTAGAATGTATCAAAAAAAGCACCGACTCGGTG
UNS1_Scaffold-R	GAGAATGGATGCGAGTAATGAAAAAAAGCACCGACTCGGTG
UNS1_MtU6-F	CATTACTCGCATCCATTCTCATGCCTATCTTATATGATCAATGAGG
GFPg1	TCAAGCGAACCAGTAGGCTTGCTGAAGCACTGCACGCCGTGTTTTAGAGCTAGAAATAGC
GFPg2	TCAAGCGAACCAGTAGGCTTGGGCGAGGAGCTGTTCACCGGTTTTAGAGCTAGAAATAGC
SlEZ2g1	TCAAGCGAACCAGTAGGCTTGGCGTCGGATAACTCCCTGTGTTTTAGAGCTAGAAATAGC
SlEZ2g2	TCAAGCGAACCAGTAGGCTTgCAGGGCTCCATATCTGCATGTTTTAGAGCTAGAAATAGC
GFPseq-F	GCTCCTGGACGTAGCCTTC
GFPseq-R	GGAGAGGACAGGCTTCTTGA
SlEZ2seq-F1	CCGTCAAAATCCGATTCCTA
SlEZ2seq-R1	CAGTGTAACATTGGAAAACAATCA
SlEZ2seq-F2	GGTCTTCCGACAGATGTTTCA
SlEZ2seq-R2	AAGCAGGACGCTGAATTGAT

Suppplemental table 2: average seed weight in cGFP and gSlEZ2 fruit

		seed no.	no. of fruits	total dry seed weight (gr)	avg seed weight (mg)
cGFP	cGFP-i	131	3	0.3782	2.887
	cGFP-ii	69	3	0.1943	2.816
	cGFP-iii	100	3	0.3086	3.086
gSlEZ2	gSlEZ2-i	109	2	0.3125	2.867
	gSlEZ2-ii	34	1	0.103	3.029
	gSlEZ2-iii	99	3	0.29	2.929
